# Exposure to Iron Oxide Nanoparticles Coated with Phospholipid-Based Polymeric Micelles Induces Renal Transitory Biochemical and Histopathological Changes in Mice

**DOI:** 10.3390/ma14102605

**Published:** 2021-05-17

**Authors:** Mihaela Balas, Ioana Mihaela Popescu Din, Anca Hermenean, Ludmila Otilia Cinteza, Anca Dinischiotu

**Affiliations:** 1 Department of Biochemistry and Molecular Biology, Faculty of Biology, University of Bucharest, 91-95 Splaiul Independentei, 050095 Bucharest, Romania; mihaela.balas@bio.unibuc.ro (M.B.); mihaela.ioana.popescu@gmail.com (I.M.P.D.); 2 Department of Experimental and Applied Biology, Institute of Life Sciences, Vasile Goldis Western University of Arad, 86 Rebreanu, 310414 Arad, Romania; anca.hermenean@gmail.com; 3 Department of Histology, Faculty of Medicine, Vasile Goldis Western University of Arad, 1 Feleacului Street, 310396 Arad, Romania; 4 Department of Physical Chemistry, Faculty of Chemistry, University of Bucharest, 4-12 Regina Elisabeta Blvd, 030018 Bucharest, Romania; ocinteza@gw-chimie.math.unibuc.ro

**Keywords:** iron oxide nanoparticles, phospholipidic micelles, oxidative stress, kidney fibrosis

## Abstract

The renal toxicity induced by the intravenously injected iron oxide nanoparticles (IONPs) encapsulated in phospholipid-based polymeric micelles was studied in CD1 mice for 2 weeks. Two doses of 5 and 15 mg of Fe/kg bodyweight of NPs or saline solution (control) were tested, and the levels of antioxidant enzyme activities, oxidative stress parameters, and the expressions of kidney fibrosis biomarkers were analyzed. The enzymatic activities of superoxide dismutase, catalase, glutathione peroxidase, glutathione-S-transferase, glutathione reductase, and glucose-6-phosphate dehydrogenase in the kidney were significantly decreased compared to the control in the first 3 days followed by a recovery up to 14 days. Concomitantly, a significant increase in lipid peroxidation (malondialdehyde) levels and a decrease in protein thiol groups were recorded. Moreover, increases in the expressions of T cell immunoglobulin and mucin domain 1 (TIM-1) and transforming growth factor-β (TGF-β) were observed in mouse tissue samples in the first week, which were more pronounced for the higher dose. The results suggested the role of oxidative stress as a mechanism for induced toxicity in mice kidneys after the IV administration of IONPs encapsulated in phospholipid-based polymeric micelles but also the capacity of the kidneys’ defense systems to revert efficiently the biochemical modifications that were moderate and for short duration.

## 1. Introduction

Engineered iron oxide nanoparticles (IONPs) have been developed for a wide range of biomedical applications, including imaging, therapy, or theranostics [1,2,3]; thus, the understanding of their biokinetics and toxicity is of fundamental importance for their clinical translation. The IONPs introduced to the bloodstream are recognized and up-taken by macrophages of the mononuclear phagocytic system (MPS), residing in the dedicated filtration organs (i.e., liver, kidney spleen, and lung), ultimately resulting in the excretion either by renal or hepatobiliary elimination [4,5]. Previous studies have shown that more than 80% of the intravenously (IV) injected IONPs accumulated into the liver, 10% into the spleen, while less than 2% were found in the kidneys, lungs, and heart following up to 14 days of exposure [6]. Thus, many organs might be exposed to the potentially deleterious effects of IONPs through different pathways. The toxic effects of NPs depend on their physicochemical characteristics such as size, shape, surface charge, and surface coating. Usually, upon entering the bloodstream, proteins are adsorbed on the surface of NPs (by opsonization), altering their surface charge and hydrodynamic size. It has been reported that NPs smaller than the glomerular filtration size limit (∼5.5 nm) undergo renal elimination by the kidneys and leave the body via urine. The NPs larger than 5.5 nm might be disintegrated or retained by Kupffer cells before undergoing hepatobiliary elimination [7]. To accomplish the requirements for diagnosis and therapeutic use, in addition to biocompatibility, NPs should present a size in the range of 5–100 nm, allowing a maximal blood circulation time, as well as permeability and retention effects [8]. For this purpose, NPs should be specially designed by using appropriate surface coatings.

Among the most advanced clinical applications in the diagnostics and treatment of human diseases, polymeric micelles are an important example of the new generation of coatings. Polymeric micelles are nanoscale-sized particles (5–200 nm) composed of poly(ethylene glycol)-based amphiphilic block copolymers, which consist of two parts: the hydrophobic part on the inside (core) and the hydrophilic part on the outside (shell). Most importantly, these polymeric micelles have low toxicities and can be removed by renal filtration [9,10]. Besides biocompatibility, the hydrophilic shell provides several advantages to NPs such as their stabilization in aqueous solution or biological media, the improvement of the dispersity of the particles, the prevention of their aggregation and opsonization, and the reduction of macrophage uptake, which enables prolonged blood circulation [11,12].

Several examples of the improvement of IONPs properties after encapsulation in polymeric micelles are available in the scientific literature. Starmans et al. (2015) [13] reported that IONPs encapsulated in lipidic micelles allow significantly more sensitive magnetic particle imaging (MPI) than current commercially available IONPs formulations and more sensitive magnetic resonance imaging (MRI) detection due to their strong T2 effect [14]. Moreover, micelles-coated IONPs showed heating properties such that when they were combined with a chemotherapeutic agent, a targeting ligand could be used for localized, triggered drug delivery [15].

However, under some circumstances, the surface coating can be degraded, exposing internal IONPs to biological systems, which probably cause undesired adverse effects. Understanding the in vivo behaviors of IONPs is crucial for the assessment of their potential health risk. Yet, the off-target impact of IONPs exposure is largely unknown because the mechanisms of IONPs interactions with the healthy cells and tissues and how they affect the body systems are not well understood [16]. Although there is an increasing number of studies devoted to understanding the interaction of IONPs with biological systems, published data on the biodistribution and toxicity of IONPs are not consistent, which might be due to the variation in IONPs characteristics and experimental conditions. Thus far, data on the in vivo toxicity of IONPs encapsulated in polymeric micelles have been reported in mice lung [17], spleen [18], and liver [14].

Our study focused on the investigation of the short-term toxicity induced in CD1 mice kidneys by the IV administration of IONPs encapsulated in phospholipid-based polymeric micelles.

We show here how these NPs might affect the modulation of antioxidant enzyme activities and levels of oxidative stress markers, and the expression of kidney fibrosis biomarkers up to 14 days after administration.

## 2. Materials and Methods

### 2.1. IONPs Encapsulated in Phospholipid-Based Polymeric Micelles

The IONPs encapsulated in phospholipid-based polymeric micelles used in this study were designed and prepared as previously described [17]. Hydrophobic magnetite NPs stabilized with oleic acid were synthesized using a solvothermal method with minor modifications [19]. The encapsulation into the polymeric micelles was performed using stable 1,2-distearoyl-sn-glycero-3-phosphoethanolamine-N-[methoxy (poly(ethylene glycol))-2000] (ammonium salt) (DSPE-PEG) and the dry film hydration procedure. The encapsulation of IONPs in the phospholipid micelles was obtained by mixing the NPs with lipids during the micelle preparation. Finally, the suspension was filtered through a Millex filter (0.22 µm diameter, Merck KGaA, Darmstadt, Germany) for sterilization.

The obtained NPs were monodispersed (polydispersity index of 0.086) with an average size of 12.5 nm, and the average size of the micelle-encapsulated IONPs was 21.5 nm, slightly higher than the size of unloaded (empty) micelles prepared from phospholipid polymeric derivative DSPE-PEG [17]. The zeta potentials of unloaded (−30.1 mV) and loaded micelles (−20.7 mV) were similar to those previously reported in the literature [19]. The NPs suspension was stable, without any visible appearance of solid magnetite precipitates or variations in the micelle size for up to three months.

### 2.2. Animals and Experimental Design

A total number of 105 CD1 mice of both sexes (weighing between 25 and 30 g) were hosted at the Animal Facility of “Vasile Goldiș” Western University of Arad in individually ventilated cages (IVC) under ad libitum feeding and watering conditions, with a controlled environment (temperature, humidity, and lighting). The administration of IONPs encapsulated in DSPE-PEG micelles was performed by a single IV injection of a 40 μL suspension in the tail vein of mice. The animals were divided into three groups. Group one was injected with 0.7% sodium chloride solution (40 μL), group 2 with a dose of 5 mg of Fe/kg body weight (bw) of NPs suspension prepared in sodium chloride 0.7%, and group 3 with a dose of 15 mg of Fe/kg bw. Each group was further divided into 5 subgroups (*n* = 7 mice/subgroup) corresponding to the exposure intervals of 1, 2, 3, 7, and 14 days. After mice euthanasia, the kidney tissues were collected according to the five predetermined time intervals. Fragments from tissue samples were cryopreserved at a temperature of −80 °C for biochemical determinations but were also fixed with paraformaldehyde and embedded in paraffin for histopathological analyzes.

All experimental procedures were performed according to Directive 2010/63/EU and national legislation (Law no.43/2014) and were approved by the Vasile Goldis Western University Ethics Committee by authorization No.32/2015.

### 2.3. Preparation of Kidney Tissue Homogenates

An amount of 0.1 g of mouse kidney tissue was homogenized (after thawing from −80 °C) in one milliliter of lysis buffer (0.1 M Tris/5 mM EDTA), pH 7.4, and was sonicated on ice with an Ultrasonic Procesor UP50H from Hielscher (Hielscher Ultrasound Technology, Teltow, Germany) at 80% amplitude, 1 cycle for 30 s, 3 times. Tissue homogenates were left for one hour at 4 °C and were then centrifuged at 10,000 rpm and 4 °C for 30 min. Finally, the supernatants were collected and aliquoted for subsequent biochemical determinations. The total protein concentration was quantified using Lowry’s method (1951) [20] and bovine serum albumin (BSA, A9647, Sigma-Aldrich, St. Louis, MO, USA) as standard.

### 2.4. Measurement of Specific Enzyme Activities

The specific activities of catalase (CAT), superoxide dismutase (SOD), glutathione peroxidase (GPX), glutathione reductase (GR), glutathione-S-transferase (GST), and glucose-6-phosphate dehydrogenase (G6PDH) were measured in kidney tissue homogenates, as previously described [17,21,22]. All the enzymatic activities were expressed as specific activities (U/mg of protein) and represented as % from controls.

### 2.5. Quantitative Determination of Reduced Glutathione (GSH) Content

The GSH concentration from kidney tissue homogenates was quantified using the Glutathione Analysis Kit (CS0260, Sigma-Aldrich, St. Louis, MO, USA), according to the manufacturer’s instructions. Briefly, the tissue extracts were deproteinized with 5% sulfosalicylic acid (SSA) (1:1) and centrifuged at 10,000 rpm for 30 min and 4 °C. Then, a volume of 10 μL of each supernatant obtained was homogenized with 150 μL of working mixture containing 1.5 mg/mL of 5.5′-dithiobis-(2-nitro benzoic acid) prepared in 100 mM of potassium phosphate, at a pH of 7.0, and with 1 mM of EDTA, and it was incubated for 5 min at room temperature. The absorbance was read at 412 nm using a microplate reader. The results were expressed in nmoles/mg protein and represented as % from controls.

### 2.6. Lipid Peroxidation Assay

The level of malondialdehyde (MDA), a marker of polyunsaturated fatty acid peroxidation, was measured by a fluorometric method. [23]. First, a volume of 200 μL of tissue extract, appropriately diluted, was mixed with 700 μL of 0.1 N HCl and was incubated for 20 min at room temperature. Secondly, a volume of 900 μL of 0.025 M thiobarbituric acid (TBA) was added and the mix was incubated again at 37 °C for 65 min. Finally, a volume of 400 μL of 0.1 M Tris-HCl buffer/5 mM of EDTA, pH 7.4, was added, and the relative fluorescence units (RFU) were recorded with a spectrofluorimeter at λex = 520 nm and λem = 549 nm. The MDA concentration in samples was estimated using a calibration curve ranging from 0 to 5 μM of 1,1,3,3-tetramethoxypropane (108383, Sigma-Aldrich, St. Louis, MO). The results were expressed in nmoles/mg protein and were represented as % from controls.

### 2.7. Analysis of Oxidative Modifications of Proteins

a. *The protein thiol groups (P-SH)* concentration was estimated in kidney tissue extracts using 4,4′-dithiodipyridine (DTDP) following the method adapted by Riener et al. [24]. In this method, P-SH reacts with DTDP and results in 4-pyridyldithio-derivatives (R-S-S-Pyr), which can subsequently react with other P-SH groups, leading to the formation of symmetrical products with disulfide bridges (R-S-S-R). Briefly, a volume of 100 μL of the tissue extract was deproteinized with 100 μL of trichloroacetic acid (TCA) 20%, and the mixture was left on ice for 10 min. After centrifugation of the samples for 10 min at 10,000 rpm and 4 °C, the supernatant was carefully removed, and the remaining pellet was completely dissolved in 20 μL of 1 M NaOH solution. Then, a volume of 730 μL of 0.4 M Tris buffer, pH 9.0, and a volume of 30 μL of 4 mM DTDP solution were added, and the mix was incubated in darkness for 5 min at room temperature. The absorbance was read at 324 nm using a UV/VIS spectrophotometer (PerkinElmer, Waltham, MA, USA). The concentration of P-SH was calculated using a calibration curve with N-acetyl cysteine (0–100 μM), and the results were expressed in nmoles/mg protein.

b. *The advanced oxidation protein products (AOPP)* level was measured using a colorimetric method adapted by Witko-Sarsat et al. [25]. Briefly, a sample volume of 200 μL appropriately diluted was mixed with 10 μL of KI and incubated for 5 min at room temperature in a 96-well plate. Then, a volume of 20 μL of glacial acetic acid was added to this mixture and was stirred for 30 s at room temperature. The absorbance was read at 340 nm. The AOPP concentration was calculated using a calibration curve with chloramine-T (0−100 μM) and the results were expressed in μmoles chloramine/L.

### 2.8. Immunohistochemistry (IHC) Staining for TGF-Beta and TIM1

Immunohistochemical evaluation of kidney tissue sections was performed using the System Max Polymer Detection kit (NovoLink Kit, Novocastra^®^, Newcastle, UK). The 5 µm thick sections were deparaffinized in toluene and rehydrated. After blocking endogenous peroxidase with 3% H_2_O_2_ for 10 min, the sections were incubated with a blocking solution from the kit and were then incubated at 4 °C overnight with a 1:100 dilution of primary antibody for TGF-β (Santa Cruz Biotechnology Inc., sc-130348) and TIM1 (Thermo Scientific, PA5-20244, Carlsbad, CA, USA). The detection was performed using a polymer system (NovoLink max Polymer detection system, Novocastra^®^ Leica Biosystems) and DAB (3,3′-diaminobenzidine, Novocastra Leica Biosystems) as a chromogenic substrate. Nuclei labeling with hematoxylin was performed before dehydration and slide mounting. The examination and the image acquisition were performed using an Olympus BX43 microscope. Quantification of IHC images was performed with ImageJ software (v 1.51k, NIH, Bethesda, MD, USA) using the Particle analysis function (i.e., the total of colored pixels of a biomarker). First, the IHC image was converted into 8-bit (Image/Type/8-bit) and appeared in shades of gray. After that, a threshold was set (Image/Adjust/Threshold). As a consequence, regions that were marked in brown (IHC staining) appeared in red. Using the up and down cursor on the dialog box, the red color was adjusted to overlap the regions that were colored brown in the original photo (it was easier to look at the unmodified photo in parallel). The threshold was compared with that from an image of the control (basic threshold, which corresponds to the coloration of the nuclei). After the proper threshold was chosen, “Analyze/Analyze particles/Summarize” were selected from the bar menu. Finally, the results were retrieved as % Area (percentage of stained area).

### 2.9. Statistical Analyses

The data were analyzed using GraphPad Prism software (version 6, Inc., La Jolla, CA, USA) and were expressed as mean values ± standard deviation (SD). The values obtained on each animal group were compared by the one-way ANOVA test followed by a Bonferroni post-hoc test. The results were considered statistically significant at a probability value (*p*) less than 0.05.

## 3. Results

### 3.1. Variation in Antioxidant Enzyme Activity in Mice Kidneys

**Figure 1:**
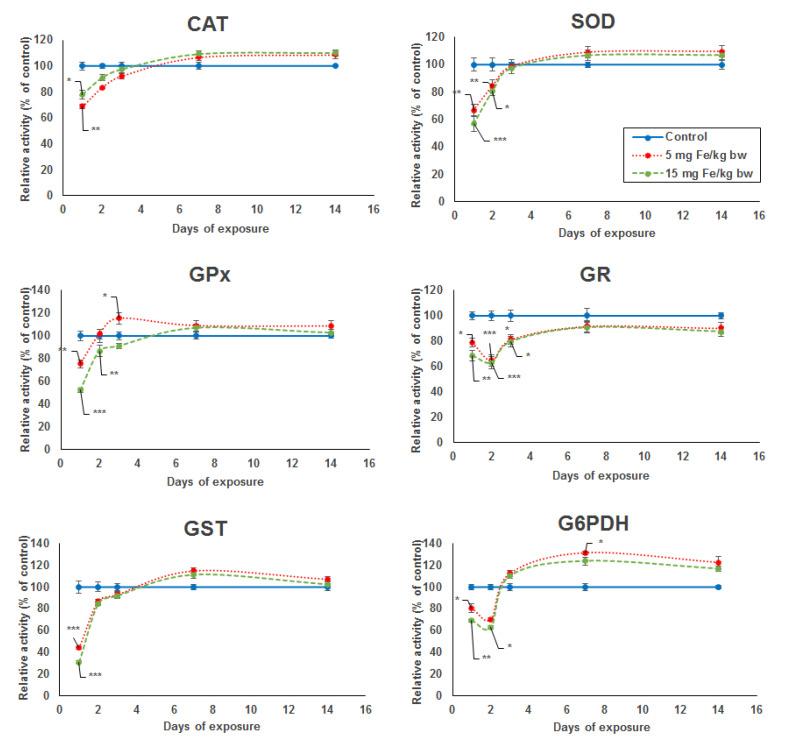
Variation in enzymatic activity of CAT, SOD, GPx, GR, GST, and G6PDH from kidneys’ tissue extracts of CD1 mice treated with 5 and 15 mg of Fe/kg body weight (bw) of IONPs encapsulated in phospholipid-based micelles at 1, 2, 3, 7, and 14 days post-administration. The values refer to the mean ± standard deviation (SD) and are expressed as percentages of control; the results were considered statistically significant when * *p* < 0.05; ** *p* < 0.01; *** *p* < 0.001 versus the control group.

The activity level of five key enzymes of the antioxidant defense system (SOD, GPx, GST, GR, and G6PDH) showed a significant dose-dependent decrease after the first day of exposure compared to the control. In the case of CAT activity, the decrease was more pronounced for the lower dose by 32% compared with the higher dose by 23% (Figure 1). A major decrease in enzymatic activity was noticed for SOD, GPx, and GST by 44%, 48%, and 70%, respectively, in the kidney of mice treated with the higher dose. For the same dose, the specific activity level of GR and G6PDH was reduced only by 32% and 31%, respectively, but comparing with the other enzymes, the activity decreased more after the second day by 37% and 38%, respectively. The enzymatic activity levels of kidney CAT, SOD, GPx, and GST elevated from the second to the seventh day from the administration of micelle-encapsulated IONPs in mice exposed to both doses, reaching the level observed in the control group, which was somewhat maintained until day 14 of experiment. Starting at day 3 until day 14, an increase in enzymatic levels was also observed for GR and G6PDH, but, only for the last one, the activity surpassed the level of the control group, and even a significant increase of 31% was noticed in the kidneys of mice treated with 5 mg of Fe/kg bw after the seventh day.

### 3.2. Expression of Oxidative Stress Markers

**Figure 2:**
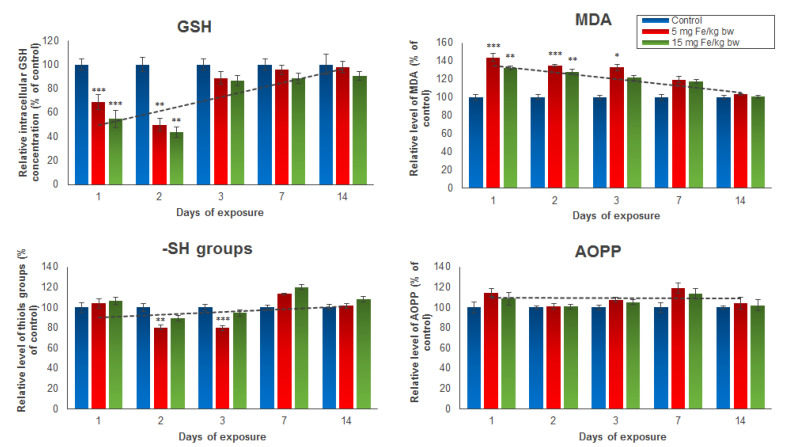
The levels of GSH, MDA, -SH groups, and AOPP from mice kidney tissue extracts, after 1, 2, 3, 7, and 14 days from the administration of 5 and 15 mg of Fe/kg bw of IONPs encapsulated in phospholipid-based micelles. The values are calculated as the mean ± standard deviation (SD) and are expressed as percentages related to the control group; the results were considered statistically significant when * *p* < 0.05; ** *p* < 0.01; *** *p* < 0.001 versus the control group. The dotted line indicates the tendency of parameter variation.

Several oxidative stress markers such as GSH, MDA, protein thiol groups, and AOPP were analyzed post-administration of the IONPs encapsulated in phospholipid-based micelles from mice kidney tissue extracts (Figure 2). The results revealed a significant and dose-dependent reduction of intracellular GSH stores in the first two days in mice treated with both doses of NPs, but a restoration of GSH level starting with day 3 until day 14 compared to the control group was observed. For mice injected with 5 mg of Fe/kg bw NPs, a decrease in GSH concentration in kidney tissue by 32% in the first day and 51% in the second day was noticed. For mice injected with 15 mg of Fe/kg bw NPs, the GSH levels were by 46% and 57%, respectively, lower than those registered in the control group. In correlation with these results, the level of MDA, an end-product of lipid peroxidation, was significantly elevated in the first three days post-administration, and then started to drop until day 14 when it reached the control level. The increase in MDA production in the first 3 days was slightly higher in mice injected with 5 mg of Fe/kg bw of IONPs encapsulated in phospholipid-based micelles (by 43%, 34%, and 32%, respectively) compared with that observed in mice injected with 15 mg of Fe/kg bw (by 32%, 28%, and 21%, respectively). The thiol protein groups and AOPP levels were measured to estimate the protein oxidation amplitude. The only significant change from the control level was observed in the case of the thiol protein groups level found in proteins after the second and third days from administration of the 5 mg dose of Fe/kg bw, which decreased by 20% and 21%, respectively, versus the control. The AOPP level was not affected by both doses of IONPs encapsulated in phospholipid-based micelles during the experiment.

### 3.3. Immunohistochemistry Expression of TIM-1 and TGF-β

**Figure 3:**
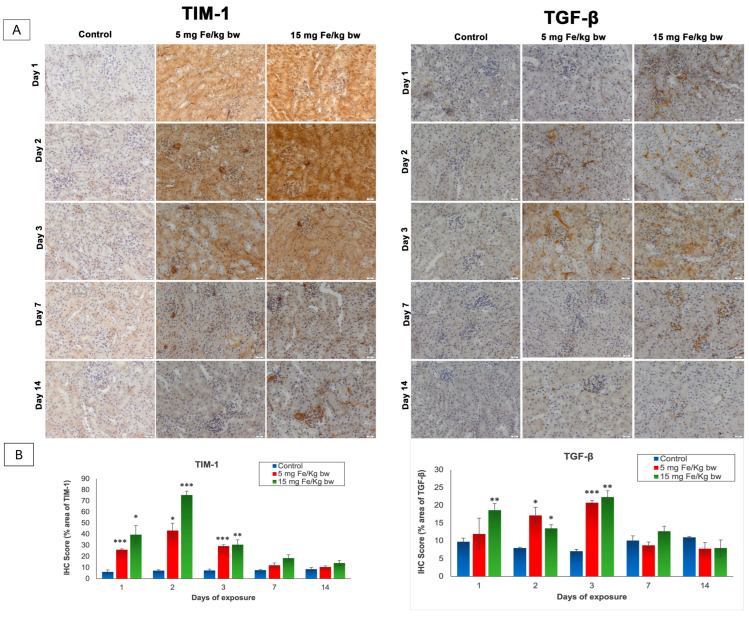
The effect of IONPs encapsulated in phospholipid-based micelles on TIM-1 and TGF-β expression and distribution in CD1 mice kidney tissue at 1, 2, 3, and 7 days post-exposure. (**A**) Immunohistochemistry (IHC) images; (**B**) quantification of IHC images. The IHC score was expressed as a percentage of the stained area. The results are calculated as the mean ± standard deviation (SD) and are considered statistically significant when * *p* < 0.05, ** *p* < 0.01, and *** *p* < 0.001 versus the control group. Scale bar: 20 μm.

The T cell immunoglobulin and mucin domain 1 (TIM-1), also known as kidney injury molecule-1 (KIM-1), and the transforming growth factor-β (TGF-β) are two molecules that play important roles in the signaling of kidney injury [26,27]. The expression of these parameters was revealed by immunohistochemistry (IHC) and was quantified with the ImageJ program (Figure 3). As shown in Figure 3, the expression of TIM-1 is absent in the control kidney sections but was present in the cortical area and mainly in the proximal tubules in the kidney section of mice injected with both doses of IONPs encapsulated in phospholipid-based micelles. Thus, from the first day post-administration, the TIM-1 expression was high, reaching the maximum level on day 2 and persisting up to day 7. After this period, TIM-1 expression was observed only in the renal glomeruli exposed to 15 mg of Fe/kg bw NPs and decreased almost to the control level in mice injected with 5 mg of Fe/kg bw NPs. Moreover, a significant increase in TGF-β expression over the control level was observed in the first day only in mice injected with a dose of 15 mg of Fe/kg bw of IONPs encapsulated in phospholipid-based micelles more exactly in the mesangial cells of renal glomeruli and the tubulointerstitial spaces compared to the other groups where the pro-fibrotic marker was not expressed. The TGF-β expression reached a maximum level at 3 days post-administration when immunopositivity was observed in the renal cortex at most tubules and renal glomeruli, for both doses. On day 7, the expression of TGF-β began to diminish, such that after 14 days, the renal sections for both groups treated were comparable to the control.

## 4. Discussions

Kidneys are vital organs in sustaining the health of the human body not only by filtering out wastes and excess water but also by maintaining homeostasis, blood pressure, temperature, and physiological pH values. As a result, the understanding of the impact of NPs exposure on kidney functions and biochemical mechanisms triggered at this level is highly important.

The present study offers a perspective regarding the toxic effects and biocompatibility of IONPs encapsulated in phospholipid-based micelles with a hydrodynamic diameter of around 21.5 nm in the mice kidney on a period of two weeks after one IV administration. According to several studies [7,28], this size did not allow NPs to cross the glomerular capillary wall and to be eliminated by renal filtration. However, after IV injection, some dissociated fragments might be eliminated from the body in the urine. Generally, the NPs with a diameter size >10 nm are taken up by the specialized macrophages from the liver (Kupffer cells) and spleen, which are the major clearance pathways for the IONPs from the bloodstream [29]. When injected in high doses, the liver and spleen macrophages can only eliminate a fraction of the IONPs from the bloodstream, and the excess IONPs accumulate in other macrophage-rich tissues such as lung and adipose tissue [30,31].

In blood plasma, iron is bound to transferrin and loosely to albumin or endogenous chelators such as citrate, malate, acetate, phosphate, and adenine nucleotides [32]. Our previous studies demonstrated the presence of iron in small depositions in the mice liver [14] and spleen tissue, up to 3 days post-administration [18], but no NPs were detected in the lung tissue [17]. However, another study reported very small quantities of iron in mice urine (0.035% of the injected dose) after administration of polyglycerol-grafted IONPs (39 nm), thus making them susceptible to renal excretion. The MRI images showed that polyglycerol-grafted IONPs accumulated rapidly in the liver and also in the kidneys within the first 6 min after administration, but they were washed out about 80 (in liver) to 110 min (in kidneys) post-injection, conceivably owing to the hydrophilic and stealth coating of the NPs [33].

Several studies revealed that iron is present in primary urine due to the post-filtration at the glomerulus level, and it is reabsorbed in the proximal tubule cells. The transferrin-bound iron is up-taken by endocytosis mediated by transferrin receptor 1, as well as cubilin and megalin receptors present in the apical domain of the membrane. The non-transferrin-bound iron is taken up by other mechanisms [34].

Along the length of nephron, iron is re-absorbed through different specific transporters such as DMT1, ZIP8, and ZIP14. DMT1 is expressed only in the cortex at the apical pole of proximal tubule cells. In addition, ZIP8 and ZIP14 transport non-transferrin-bound iron and other divalent cations [35].

In mice kidney proximal tubules, two iron regulatory proteins IRP-1 and IRP-2 are important in iron homeostasis, IRP-1 having a higher expression [36]. They are present in cytosol and sense cytosolic iron levels by binding to RNA-steam-loop motifs of mRNA transcripts of iron-responding genes. As a result, they regulate the expression of H and L chains of ferritin and other iron proteins. The two subunits of ferritin have different functions. Thus, the H subunit has a ferroxidase activity, converting Fe^2+^ to Fe^3+^, and it is an iron chelator, whereas the L subunit facilitates the nucleation and mineralization of the iron center [35].

In addition, the tubular cells present ferroportin, an iron exporter, that works together with hephaestin, a ferric oxidase, in order to export iron in circulating blood [37].

Thus, a highly important step in understanding the impact of NPs on organisms for a safe clinical translation is the discovery of a link between their absorption, distribution, metabolism, and excretion, which is possible only by tracking the effects generated by NPs through structural and biochemical modifications in the whole body.

It is well-documented that IONP-induced toxicity is mainly due to the oxidative stress mediated via reactive oxygen species (ROS) production originating from iron ions. Subsequently, after cellular internalization, inside the lysosomes, magnetite (Fe_3_O_4_) NPs could be broken down into ferrous (Fe^2+^) or ferric (Fe^3+^) ions, which could be eliminated by renal clearance or incorporated into hemoglobin [38], ferritin, hemosiderin, or transferrin [39,40] for further use in the body. Released Fe^2+^ ions can participate in the presence of hydrogen peroxide, in the Fenton reaction, producing highly reactive hydroxyl radicals. As a result, lipid and protein oxidation could occur, leading to renal cell dysfunction or cell death [41], as well as DNA damage.

The renal cells are protected against ROS by a variety of defense systems, including enzymatic antioxidant mechanisms and also non-enzymatic antioxidants [42]. Our study indicated that, in CD1 mice kidneys, all analyzed antioxidant enzyme activities (CAT, SOD, GPx, GR, GST, and G6PDH) decreased significantly and dose-dependently below the level of the control in the first-day post-administration. This might be a cause of the accumulation of dissociated iron ions on the first day. It has been shown that heavy metals deplete nonenzymatic antioxidants, including reduced glutathione and thioredoxin systems, thus allowing the accumulation of ROS in kidneys [42]. Later, the accumulated iron ions were probably eliminated or metabolized and the decrease in enzyme activity started to diminish, except those of GR and G6PDH, which decreased even more on the second day post-administration.

The decrease in enzyme activities might also be a consequence of the ROS excess caused by an increase in blood pressure (via angiotensin II) and/or initiation of inflammatory processes (via pro-inflammatory cytokines), which is known to stimulate the production of superoxide anions and hydrogen peroxide in the kidneys through the nicotinamide adenine dinucleotide phosphate (NADPH) oxidases and mitochondria dysfunction [42]. In renal cells, the NADPH oxidases are localized in the plasma membrane, but one of the isoforms (NOX4) was also found in the mitochondria of rat kidney cortex, which suggests that generated superoxide anions could directly increase the physiological mitochondrial generation of ROS [43]. The macrophages from the circulation that enter the kidney could also release pro-inflammatory cytokines due to the activation of inflammasomes induced by ROS [44]. For example, Hanini and colleagues found that IV-injected maghemite γ-Fe_2_O_3_ NPs (0.8 mg/kg bw) caused inflammation in rat kidneys indicated by eosin-positive infiltrated cells [45].

SOD catalyzes the dismutation of the highly reactive superoxide anion to hydrogen peroxide, which is scavenged by CAT or GPx. In the kidney, the total SOD activity is represented by cytosolic and extracellular Cu, Zn-SOD, and mitochondrial Mn-SOD ones. A long time ago [46], it was shown that Fe-substituted Mn-SOD presented a lower activity than Mn-SOD did. As a result, in the first two days post-exposure, the total SOD activity decreased compared to the control. Thus, the reduction in total SOD activity might be responsible for the decrease in CAT and GPx activities in mice kidneys, generating reduced capacities of these enzymes to scavenge hydrogen peroxide. The dysregulation of activities of these enzymes might result in the accumulation of hydrogen peroxides, which can then react with transition metals to subsequently form highly reactive and damaging species, such as the hydroxyl radical [42]. Moreover, increased hydrogen peroxide levels might inhibit SOD activity [47].

The antioxidant defense system depends on the production of NADPH to maintain the cellular redox balance [48]. The NADPH is mainly produced by the G6PDH catalyzed reaction in the framework of the pentose phosphate pathway. Therefore, G6PDH might affect superoxide anion production via NADPH oxidase, which is a key mediator of the response to the increased angiotensin II level [49].

In this study, we found that the G6PDH activity decreased significantly in the first two days post-administration of IONPs. According to a previous study, cyclic adenosine monophosphate (cAMP) could decrease the G6PDH activity both by decreasing G6PDH gene transcription and by posttranslational modification of the existing protein [48]. Rosa and colleagues showed that compounds that increase cAMP levels led to a decrease in G6PDH activity from rat peritoneal macrophages [50]. In renal compartments, cAMP can be formed by the pancreatic-hepatorenal cAMP-adenosine pathway that can serve as an endocrine link between the pancreas, liver, and kidney [51], possibly contributing to renal toxicity. Probably, a decreased G6PDH activity and, as a result, decreased NADPH level led to the inhibition of GR activity, consequently decreasing the GSH levels. Taking this into account, the activity of GST that has, as one of the substrates, GSH, also decreased. Furthermore, it was shown that NADPH is necessary for the maintenance of the active form of CAT [52]. Nevertheless, starting with the third day, an increase in the G6PDH activity over the level of control was noticed, explaining the restoration of all the enzyme activities.

Likewise, the GST activity dramatically decreased on the first day post-administration for both doses of NPs. This enzyme was found in epithelial cells of the kidney proximal and distal tubules [53] and contributed to the reduction in oxidative stress by the inactivation of toxic hydrophobic and electrophilic substrates by conjugation with the reduced form of glutathione (GSH). This, together with the oxidized (GSSG) form, represents the main intracellular thiol redox system in cells. The GR catalyzes the reaction that converts GSSG to GSH in an NADPH-dependent manner to maintain a high intracellular GSH:GSSG ratio and protects cells against oxidative damage. In this study, the activity of GR was decreased significantly up to the third day post-administration, which might explain the reduction in GSH level in the first two days after NP injection.

The elevation in G6PDH activity from the third-day post-administration was also in accordance with the increase in the cellular GSH level in the same period.

Our data regarding the GSH level in the kidneys of NPs-injected mice matched with those obtained in the case of human renal cell exposure to metal oxide nanoparticles [54].

The decrease in antioxidant system activity at the beginning of the experiment was also reflected in the level of lipid peroxidation products (MDA), which significantly increased in the first 3 days post-administration in mice injected with 5 mg of Fe/kg bw NPs and in the first 2 days in mice injected with 15 mg of Fe/kg bw. Later, the level of MDA returned to that of the control. Most probably, the GPx and GST contributed to the diminishing of the MDA level in the mice kidney. Our results were similar to those obtained by Lei and collaborators (2013) [55] who injected IONPs coated in OA-Pluronic (a coating consisting of oleic and Pluronic acid) in Dawley rats at a dose of 10 mg of Fe/kg bw, and they showed that the level of lipid hydroperoxides in rat kidneys increased with a maximum at 3 days, returning to normal after 3 weeks. Furthermore, our study indicated the occurrence of protein oxidations in the second and third-day post-administration by a significant decrease in protein thiol groups level in the mice kidneys. However, no significant change in AOPP level was observed compared to the controls.

In accordance with the data presented above, we identified a highly increased expression of TIM-1 (a marker of renal injury) in kidney tissue in the first 3 days after NPs injection. Although undetectable in normal kidneys, TIM-1 was found to be abundantly expressed in the acute renal injury resulting from ischemia, hypoxia, toxicity, or some renal tubular interstitial, and polycystic kidney disease [27]. There is no report in the literature relating TIM-1 expression to the renal toxicity of NPs, but our findings are in agreement with the toxicological results obtained from other renal toxicants [56,57]. The high expression of TIM-1 was localized in the renal cortical area and tubules, starting with the first day after NP injection, with a maximum at 2 days post-injection after which the expression gradually diminished until day 14. These findings might indicate that some renal morpho-functional alterations occurred.

The administration of IONP encapsulated in phospholipid-based micelles also led to the activation of the main pro-fibrotic marker TGF-β in kidney tissue in the first 3 days [58]. This observation is important, especially considering the involvement of TGF-β in the generation of renal fibrotic pathology, which arises as a result of its direct involvement in the regulation of cell proliferation [59], cell death [60], and the synthesis and degradation of extracellular matrix [61,62]. However, emerging evidence suggests that TGF-β is capable of inducing not only profibrotic effects but also protective effects by mechanisms that include the inhibition of inflammation and the induction of autophagy [26].

In our study, the TGF-β expression increased significantly in kidneys of NP-injected mice from day one, reaching a maximum increase after 3 days post-administration of both doses of NPs, where immunopositivity was observed in the renal cortex at the level of most renal tubules and renal glomeruli. After this period, the expression of TGF-β became similar to the control. We assume that TGF-β production in the kidneys of mice injected with NPs might play a dual role in the maintenance of balance through this negative self-regulation of profibrotic effects, which might be important for tissue homeostasis.

Summarizing, we showed that toxic effects were induced in CD1 mice kidneys in the first 3 days after administration, and they were, in general, proportional to the administrated doses of IONP encapsulated in phospholipid-based micelles: 5 and 15 mg of Fe/kg bw. We assumed that the toxicity in kidney tissue might not be related to a direct NPs interaction; what is more likely is the consequence of an integrated response of the body systems to iron accumulation. The toxic effects were most likely caused by iron ions from metabolized NPs brought by circulating macrophages in the kidneys or by inflammatory processes induced as a consequence of the alterations induced in the kidneys or other organs. The transient increase in kidney enzyme levels or increase in oxidative stress does not seem to be high enough to damage the kidney. The generation of lipids and protein oxidation products was limited and managed by the antioxidant kidney system. One-week post-administration, the recovery of the levels of all parameters was registered until the end of the experimental period of 14 days. The results indicated the efficiency of the antioxidant system to counteract the oxidative stress effects induced by the IV administration of IONPs encapsulated in phospholipid-based micelles in mice kidneys.

## 5. Conclusions

This is the first study concerning the toxicity produced during 14 days by the IV administration of IONPs encapsulated in phospholipid-based micelles in the kidney of mice.

According to our data, the oxidative stress generated post-exposure to these NPs represented an important mechanism of toxicity in CD1 mice kidneys.

The most significant biochemical modifications were observed in the first 3 days post-administration when the most severe immunohistochemical changes also occurred. The level of oxidative stress markers changed transiently, returning close to the control level after 1 week. Taking into account all data, we can thus state that the antioxidant defense system of mice kidneys counteracted efficiently the oxidative stress induced by exposure to this type of NPs. The inflammatory response in the kidney was minor and of short duration.

Even though all parameters recovered completely, showing good biocompatibility of these NPs, the adverse effects induced in the first week might not be the same in patients who suffer from other conditions, thus posing a threat to their lives. These studies should help to further optimize the formulation and to enhance the efficiency of our IONPs encapsulated in phospholipid-based micelles for imaging and drug delivery applications.

## Data Availability

Data sharing not applicable.
