# Correction: Balas et al. Exposure to Iron Oxide Nanoparticles Coated with Phospholipid-Based Polymeric Micelles Induces Renal Transitory Biochemical and Histopathological Changes in Mice. *Materials* 2021, *14*, 2605

**DOI:** 10.3390/ma18194526

**Published:** 2025-09-29

**Authors:** Mihaela Balas, Ioana Mihaela Popescu Din, Anca Hermenean, Ludmila Otilia Cinteza, Anca Dinischiotu

**Affiliations:** 1 Department of Biochemistry and Molecular Biology, Faculty of Biology, University of Bucharest, 91-95 Splaiul Independentei, 050095 Bucharest, Romania; mihaela.balas@bio.unibuc.ro (M.B.); mihaela.ioana.popescu@gmail.com (I.M.P.D.); 2 Department of Experimental and Applied Biology, Institute of Life Sciences, Vasile Goldis Western University of Arad, 86 Rebreanu, 310414 Arad, Romania; anca.hermenean@gmail.com; 3 Department of Histology, Faculty of Medicine, Vasile Goldis Western University of Arad, 1 Feleacului Street, 310396 Arad, Romania; 4 Department of Physical Chemistry, Faculty of Chemistry, University of Bucharest, 4-12 Regina Elisabeta Blvd, 030018 Bucharest, Romania; ocinteza@gw-chimie.math.unibuc.ro

In the original publication [1], there was a mistake in the published form of Figure 3. The corrected version of Figure 3 appears below. The authors state that the scientific conclusions are unaffected. This correction was approved by the Academic Editor. The original publication has also been updated.

## Figures and Tables

**Figure 3:**
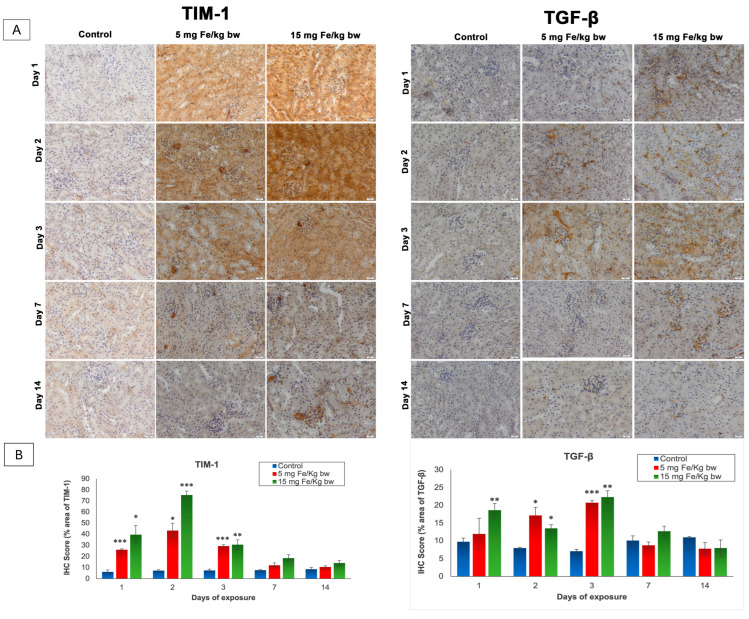
The effect of IONPs encapsulated in phospholipid-based micelles on TIM-1 and TGF-β expression and distribution in CD1 mice kidney tissue at 1, 2, 3, and 7 days post-exposure. (**A**) Immunohistochemistry (IHC) images; (**B**) quantification of IHC images. The IHC score was expressed as a percentage of the stained area. The results are calculated as the mean ± standard deviation (SD) and are considered statistically significant when * *p* < 0.05, ** *p* < 0.01, and *** *p* < 0.001 versus the control group. Scale bar: 20 μm.
